# Cap-specific terminal N6-methyladeonsine methylation of RNA mediated by PCIF1 and possible therapeutic implications

**DOI:** 10.1016/j.gendis.2023.101181

**Published:** 2023-11-23

**Authors:** Hui Zeng, Yidong Wu, Xinghua Long

**Affiliations:** a Center of Clinical Laboratory, Hangzhou Ninth People's Hospital, Hangzhou, Zhejiang 311225, China; b Department of Laboratory Medicine, Zhongnan Hospital of Wuhan University, Wuhan, Hubei 430071, China

**Keywords:** Cancer, m6Am, PCIF1, RNA modification, Virus infection

## Abstract

Posttranscriptional RNA modification is an important mode of epigenetic regulation in various biological and pathological contexts. N6, 2′-O-dimethyladenosine (m6Am) is one of the most abundant methylation modifications in mammals and usually occurs at the first transcribed nucleotide. Accumulating evidence indicates that m6Am modifications have important roles in RNA metabolism and physiological and pathological processes. PCIF1 (phosphorylated C-terminal domain interacting factor 1) is a protein that can bind to the phosphorylated C-terminal domain of RNA polymerase II through its WW domain. PCIF1 is named after this binding ability. Recently, PCIF1 has been identified as a cap-specific adenine N6-methyltransferase responsible for m6Am formation. Discovered as the sole m6Am methyltransferase for mammalian mRNA, PCIF1 has since received more extensive and in-depth study. Dysregulation of PCIF1 contributes to various pathological processes. Targeting PCIF1 may hold promising therapeutic significance. In this review, we provide an overview of the current knowledge of PCIF1. We explore the current understanding of the structure and the biological characteristics of PCIF1. We further review the molecular mechanisms of PCIF1 in cancer and viral infection and discuss its therapeutic potential.

## Introduction

In 1957 Francis Crick proposed the “central dogma of molecular biology”, which states that biological information is sequentially transferred from DNA to RNA to protein.^1^ RNA, which refers in particular to messenger RNA (mRNA), serves as a blueprint of the genetic information encoded in DNA and functions as a template for protein translation. The flow of genetic information between the different steps is considerably complex. In consideration of biodiversity, the coding capacity of RNA extends beyond the sequence of the four standard nucleotides. This complexity is partially driven by different chemical RNA modifications, collectively called epitranscriptome. The concept of epitranscriptome was initially introduced with the transcriptome-wide mapping of N6-methyladenosine (m6A), and for decades, epitranscriptome has been considered to have considerable importance as a layer of gene expression regulation.^2^^,^^3^ To date, there are about more than 170 kinds of modifications that have been identified.^4^ Capping is the first modification made to RNA polymerase II (Pol II)-transcribed RNAs.^5^ Capping is essential because 5'cap protects mRNAs from degradation by exonuclease and contributes to pre-mRNA splicing, mRNA transport, and translation initiation.^6^^,^^7^ 5'cap is added to an mRNA co-transcriptionally in the nucleus as soon as the first 25–30 nucleotides are incorporated into the nascent transcript.^5^^,^^8^ Generally, the m7GpppN structure is called cap0 and the m7GpppNm and m7GpppN1mN2m structures, wherein Nm is a nucleotide with methylation at the ribose O2 position, are named cap 1 and cap 2, respectively.^8^ The predominant 5′cap structure in mammals is m7GpppNm. When the first transcribed nucleotide is modified by 2′-O-methyladenosine (Am), it can undergo a further N6 methylation to form the m7Gpppm6Am structure.^9^

Since the first nucleotide in an mRNA transcript always contains a 2′-O-methylation modification, either Am or m6Am can be the first nucleotide. In HEK293T cells, 92% of 5′-capped mRNAs contain m6Am and 8% contain Am.^10^ Early studies revealed that m6Am can be detected in up to 30% of mRNA caps.^11^^,^^12^ Recent studies have shown that m6Am percentage in mRNA species varies according to species, tissue, and cell type, ranging from 10%–50%.^13^, ^14^, ^15^ The importance of mRNA 5′cap and the high percentage of m6Am across cellular mRNAs imply the great importance of m6Am. m6Am may affect the fate of a large subset of the transcriptome.

m6Am modification was identified shortly after the discovery of m6A in 1975.^12^ The enzyme responsible for the m6Am formation in the 5′cap was purified from HeLa cells in 1978.^11^ Different from the internally localized m6A, m6Am was originally discovered at the 5′end of mRNA transcripts, and its “writer” phosphorylated C-terminal domain (CTD) interacting factor 1 (PCIF1), was only recently identified.^10^^,^^15^, ^16^, ^17^ As the only 5′cap m6Am writer, PCIF1 plays important roles in various types of physiological and pathological processes. Studying PCIF1 is expected to reveal promising insights and facilitate the study of RNA modifications.

## The overview of m6Am

The N6,2-O-dimethyladenosine (m6Am) is a modification found in the first transcribed nucleotide adjacent to the RNA methylguanosine cap. It is prevalent in eukaryotic cells, especially those of higher eukaryotes, whereas m6A exists in all life domains.^12^^,^^18^, ^19^, ^20^, ^21^, ^22^ Moreover, unlike m6A, which is present in multiple kinds of RNAs, like mRNAs, snRNAs, rRNAs, tRNAs, lncRNAs, miRNAs, and circRNAs,^23^, ^24^, ^25^, ^26^, ^27^, ^28^ m6Am has thus far only been identified in mRNAs and snRNAs.^16^^,^^29^ The m6A modification is usually enriched around stop codons, and m6Am is understood to be located in the first transcribed nucleotide of mRNA and at position 30 in the U2 snRNA.^29^

Although the abundance of m6Am seems to be only approximately one-tenth that of m6A, the variation of m6Am is greater than that of m6A.^18^^,^^30^ m6A modification is essential for regulating the splicing, translation, stability, translocation, and the high-level structure of RNAs,^31^^,^^32^ whereas m6Am is correlated with mRNA stability and translation efficiency.^10^^,^^16^ m6Am is proven to be involved in multiple human diseases, however, dysregulation of m6Am seems to be tolerated in mice. Loss of m6Am in mice does not affect viability or fertility but results in reduced body weight.^33^

The internal m6A modification is installed by “writer” proteins, including METTL3/METTL14, METTL16, WTAP, KIAA1429, ZCCHC4, RBM15, ZC3H13, and ZCCHC4.^32^^,^^34^, ^35^, ^36^, ^37^ METTL3/METTL14, the core m6A methyltransferase complex, installs m6A at internal sites in mRNA by recognizing a specific DRACH consensus motif (where A is the methylation site, D = A, G, or U, R = A or G, and H = A, C, or U).^25^ However, different from m6A, which has multiple writers and erasers and performs different functions through different readers, only two writers and one eraser of m6Am, namely, the writer PCIF1 responsible for the N6-methylation of Am as the first transcribed nucleotide, the writer METTL4, which is responsible for the N6-methylation of U2 snRNA at position 30 to generate m6Am, and the eraser FTO, which is also an m6A eraser, have been identified to date, and no m6Am readers have been identified.^18^^,^^38^ PCIF1 introduces N6-methylation on the capped substrate by specifically recognizing the N7-methylation on m7G. The motif sequence “HMAGKD” (where A is the methylation site, H = A/C/U, M = A/C, K = G/U, and D = A/G/U) presents to be one of the criteria for *de novo* methylation by METTL4.^18^

Nowadays, m6Am can be detected by multiple methods, including m6A-seq, MeRIP-seq, miClip, m6Am-exo-seq, m6ACE-seq, m6Am-seq, and CAPturAM.^39^^,^^40^ All these methods, except for method CAPturAM, are anti-m6A antibody-dependent, and the first three methods require bioinformatic analysis for m6Am detection, m6Am-seq requires the presence of the FTO protein, but FTO demethylates both m6A and m6Am. CAPturAM is an antibody-free approach that has been recently developed for the enrichment of m6Am-containing RNAs. However, it has not yet been applied to transcriptome-wide studies. Some basic information regarding m6A and m6Am are summarized in Table 1.

**Table 1:** Some available information on m6A and m6Am modifications.

	m6A modification	m6Am modification	Ref.
Domains	Humans and other mammals, flies, plants, yeast, bacteria and viruses	Vetebrates and viruses	12, 18, 19, 20, 21, 22
RNA classes	mRNA, snRNA, rRNA, tRNA, lncRNA, miRNA, circRNA	mRNA, snRNA	16, 23, 24, 25, 26, 27, 28
Location and frequency	mRNA: Typically enriched near the stop codon, but also in the coding sequence, 3`UTR and 5`UTR	mRNA: deposit in the first transcribed nucleotide snRNA: internal RNA sites, specifically at U2 snRNA position 30	11, 29
Writers	METTL3/METTL14(mRNA), METTL16(mRNA, snRNA), WTAP(lncRNA), KIAA1429(mRNA, lncRNA, circRNA and miRNAs), RBM15(mRNA, lncRNA), ZCCHC4(rRNA), ZC3H13	PCIF1(mRNA) and METTL4(snRNA)	^32^^,^^34^, ^35^, ^36^, ^37^
Erasers	FTO and ALKBH5	FTO	14, 32
Readers	YTHDF1/2/3, YTHDC2, HNRNPC, HNRNPA2B1 and eIF3	/	^32^
Sequence preference	Mettl3: DRACH consensus motif sequence (A is the methylation site, D = A, G or U, R = A or G, and H = A, C or U)	PCIF1: preference for the 5`terminal sequence of mRNAs, but without a sequence specificity. Mettl4: HMAGKD consensus motif sequence (A is methylation site, H = A/C/U, M = A/C, K = G/U, D = A/G/U)	10, 29^,^^25^
Sequencing methods	M6A-seq, MeRIP-seq, miClip, PAm6A-seq, m6A-CLIP, m6A-REF-seq and direct RNA-seq	MeRIP-seq, miCLIP, m6am-exo-seq, m6ACE-seq, m6Am-seq, CAPturAM	^25^^,^^39^, ^40^
Function	RNA splicing, translation, stability, translocation, and the high-level structure	mRNA stability and translation efficiency U2 snRNA: regulate pre-mRNA splicing of specific pre-mRNA transcripts	10, 15, 29, 32

## The structural basis of PCIF1

Human phosphorylated CTD interacting factor 1 (hPCIF1) is a kind of protein that contains 704 amino acids.^41^ It is expressed ubiquitously in human tissues and interacts with Pol II and the transcription factor DSIF in the nucleus.^42^ It has been conserved throughout animal evolution. PCIF1 was first identified as a general repressor for the transcriptional activation via its direct and specific binding to RNA pol II and mediating by its WW domain which exhibits considerable homology to Pin1 (peptidylprolyl cis/trans isomerase).^10^^,^^42^ hPCIF1 WW domain contains amino acids from 42 to 80.^42^ The WW domain is a region that is conserved across different proteins and adopts a stable structure of three antiparallel beta strands. It facilitates interactions between different proteins and has two invariant residues that are separated by 22 amino acids. The WW domain of the full-length PCIF1 is sufficient for the interaction of PCIF1 with CTD of RNA pol II. Ser 54 in the WW domain is crucial for recognizing.^41^^,^^42^ As Ser 54 participates in the recognition of the phosphorylated Ser 5 in CTD heptapeptide, the binding affinity mostly depends on the status of Ser5 phosphorylation.^10^^,^^42^ The WW domain was found to show higher affinity for a CTD with a high phosphorylation level than for a CTD with little or no phosphorylation.

**Figure 1:**
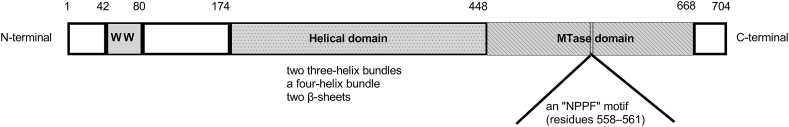
Domain organizations of hPCIF1.

In addition to the WW domain, PCIF1 has a region with a helical domain and a methyltransferase (MTase) domain. The helical domain has two groups of three helices, one group of four helices, and two groups of β-sheets. It forms a groove that has a positive charge.^10^ The MTase domain has a common structure (Rossmann fold) with a motif that can bind to S-adenosylmethionine. The m7G cap is likely to attach to the segment between the helical domain and the MTase domain and the RNA transcript downstream of the cap is likely to attach to the positively charged groove formed by the helical domain. Arg239 and Arg269 in the helical domain and Glu563 in the MTase domain are crucial for m7G cap ribose recognition. Both the N553A (asparagine 553 to alanine)^10^^,^^15^^,^^17^ and F556G^10^^,^^17^ mutations reduce the methyltransferase activity. Overall, the structure of PCIF1 forms the basis of its biological function (Fig. 1).

## Enzymatic and biological characteristics of PCIF1

**Figure 2:**
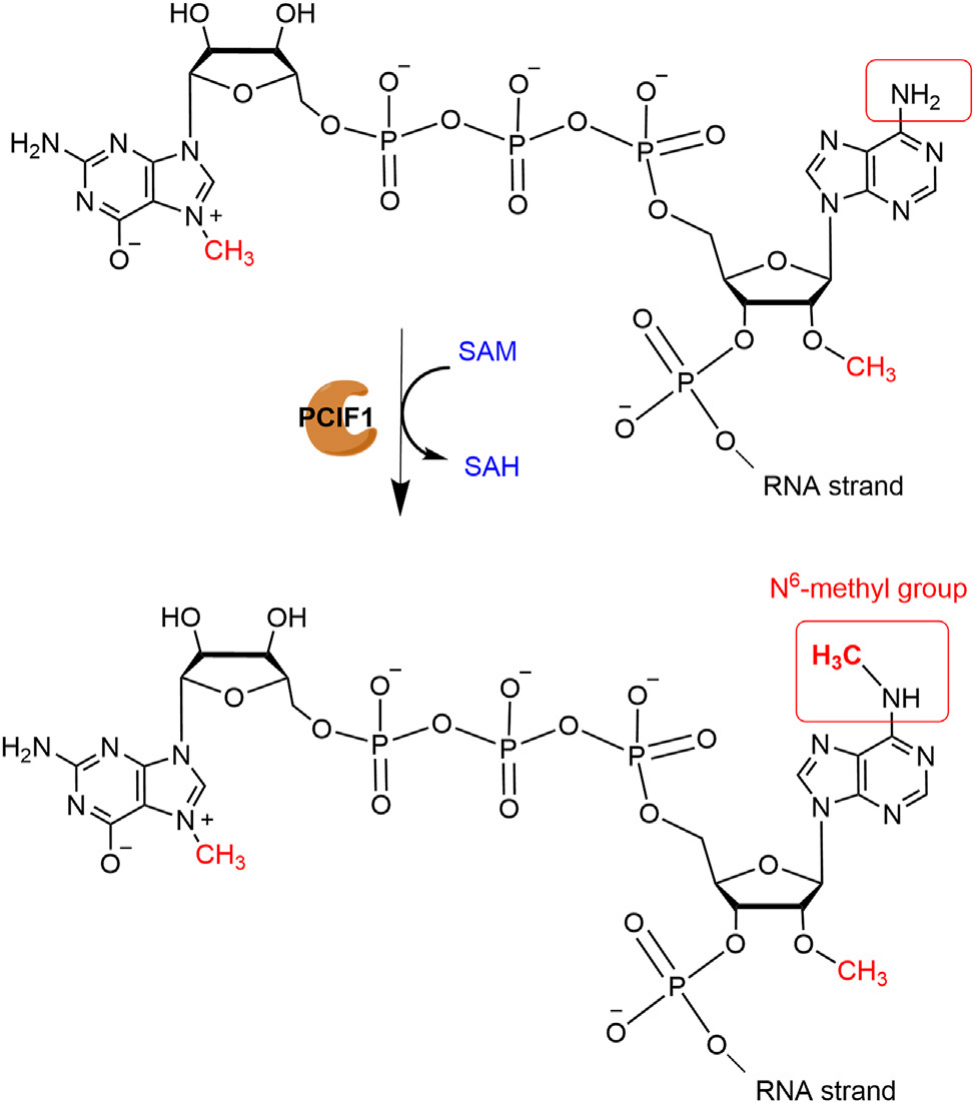
Enzymatic activity of PCIF1 in mRNA. PCIF1 specifically methylates Am-marked mRNAs and deposits the methylation group at the N6 position in the S-adenosylmethionine (SAM)-dependent manner, resulting in the generation of m6Am and S-adenosylhomocysteine (SAH).

Researchers began to notice PCIF1 long before it was identified as a methyltransferase in 2018. Previous studies^42^^,^^43^ have reported that PCIF1 negatively modulates gene expression via direct binding of its WW domain to RNA Pol II. PCIF1 was found to greatly reduce the reporter activity when its expression level was increased in 293T and Huh7 cells. The same study also showed the same effect upon a reduction in PCIF1 in HeLa cells. The reason is not clear, but the author suggested that PCIF1 might stop Pol II from being recycled by preventing the loss of its phosphate groups. This is because both the WW domain and full-length PCIF1 can prevent SCP1 from removing phosphate groups from pSer5 in the CTD *in vitro*. SCP1 is one of the FPC1-like enzymes that likes to remove phosphate groups from pSer5. PCIF1 was not known to exhibit any enzymatic activity until 2018 when Shinichiro Akichika and colleagues^10^ discovered that PCIF1 is a methyltransferase that can catalyze the addition of m6A to the 2′-O-methylated adenosine at the 5′-end of mRNAs. PCIF1 dynamically localizes to the promoter of active Pol II genes^44^ and uses S-adenosylmethionine as a methyl donor to add a methyl group to the N6 position of Am to form m6Am when Am is the first nucleotide in the RNA transcript (Fig. 2).

PCIF1 methylation activity did not exhibit strong sequence specificity for its substrates but with a preference toward substrates with a complete cap structure rather than incomplete cap or no cap.^15^^,^^16^ The reaction rate for Am modified cap analog is 3–4 times higher than that for 5′-Am oligo, but the binding affinity differs only slightly.^45^ PCIF1 preferentially targets some sequences. It exhibits maximum activity toward RNAs that start with ACG, whether they are capped^10^ or uncapped.^45^ The 2′O-ribose methylation does not affect the reaction rate, but it does affect the binding affinity. PCIF1 exhibits low activity toward adenosines that are not at the 5′ end of an RNA transcript.^45^

The mammalian MTases METTL3–14^46^, ^47^, ^48^, ^49^, ^50^ and demethylases such as FTO^51^^,^^52^ and ALKBH1^53^^,^^54^ participate in both DNA and RNA modification. The substantial methylation activity of PCIF1 on adenine was also exhibited on an ssDNA oligo but at half the level exhibited on its corresponding uncapped RNA oligo independent on a 5′ adenine or a specific sequence.^45^^,^^55^

Among capped mRNA, PCIF1 has a strong preference for substrates with an m7GpppAm cap structure, showing a 7–8 fold higher affinity for these substrates^10^^,^^15^ than for substrates without 2′ methylation on the first transcribed adenosine. The methyltransferase activity of PCIF1 is independent of RNA length, though a capped RNA dinucleotide can also be methylated^16^ and 6 nucleotides are the minimum substrate length for efficient methylation.^10^ Overall, PCIF1 is the only m6Am methyltransferase, and its activity depends on the existence of an m7G cap. Its methylation activity is independent of internal m6A. PCIF1 shows different levels of methyltransferase activity toward different substrates. Its activity is highest toward single-stranded RNA and lowest toward double-stranded DNA. It shows intermediate activity toward RNA/DNA hybrids and single-stranded DNA.

**Figure 3:**
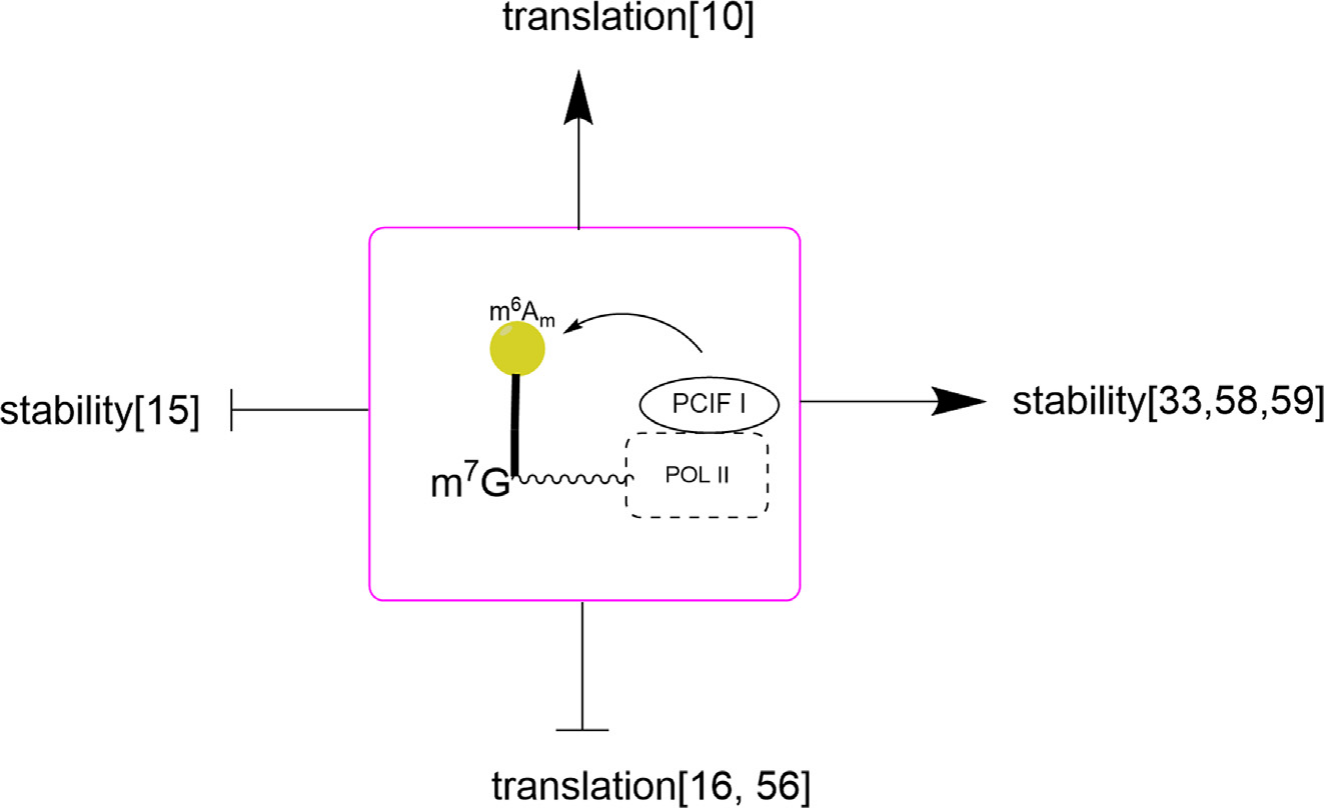
RNA polymerase II (Pol II) acts as a scaffold. PCIF1 methylated m6Am deposition changes the stability or translation of mRNA.

Since key methyltransferase enzyme PCIF1 was identified, m6Am has gradually attracted attention in the past two years. Related studies have shown that the m6Am modification mediated by PCIF1 affects the translation of the mRNA, rather than affecting the stability of mRNA.^10^^,^^16^^,^^56^ However, some studies have reported that PCIF1-mediated m6Am modification does not affect mRNA translation, but changes the stability of m6Am-modified mRNAs (Fig. 3).^15^^,^^33^^,^^57^, ^58^, ^59^ At present, there is still debate about whether the m6Am modification of mRNA 5′caps mediated by PCIF1 affects the translation or stability of mRNA. As the former approaches to study m6Am are highly dependent on the transcript annotations which may have accurate information, the identification of PCIF1 as the sole methyltransferase for cap m6Am modification enables more precise global mapping of m6Am. This is expected to pave the way for further functional and mechanistic research for m6Am.

## PCIF1 and cancer

Cancer involves abnormal cell proliferation, loss of specialization, the potential for metastasis and invasion, and impaired apoptosis. m6A modification and m6A methyltransferases have contrasting effects across cancers. PCIF1 and m6Am modification also have diverse functions in different cancers. PCIF1 was found to be a strong inhibitor of bladder cancer growth in an *in vivo* functional RNA interference screen of hundreds of cancer-related genomic changes.^60^ However, the specific function of PCIF1 has not been thoroughly studied. The role of PCIF1 in cancer was not extensively studied until its structure and function were further uncovered. Abnormal levels of PCIF1 have been found to be involved in human cancers. In 2021, a pan-cancer analysis^61^ of PCIF1 was conducted and revealed significant differential expression of PCIF1 mRNA in most tumors including colon adenocarcinoma, glioblastoma multiforme, stomach adenocarcinoma, and kidney chromophobe, compared with the corresponding adjacent normal tissues. In 2022, PCIF1 was first reported to be aberrantly overexpressed in gastric cancer and plays an oncogenic role as an m6Am methyltransferase.^56^ Mechanistically, PCIF1 modifies its mRNA target transmembrane 9 superfamily member 1 with m6Am, which reduces the translation efficiency of the mRNA target and thus its inhibitory effect on cell proliferation and invasion. The level of m6Am in serum was significantly higher in gastric cancer patients than in healthy controls. The area under the curve for distinguishing between healthy controls and gastric cancer patients was 0.647.^62^ In a novel circular RNA study,^63^ it was found that circ-ATAD1 regulates gastric cancer tumorigenesis and progression by up-regulating YY1, which directly binds to the promoter of PCIF1 and activates the transcription of PCIF1. YY1 was also found to be up-regulated in cholangiocarcinoma tissues compared with normal tissues, and high YY1 expression predicted poor prognosis.^64^ The chromatin immunoprecipitation–sequencing results suggested that in cholangiocarcinoma, six genes including PCIF1 were likely to be transcriptionally regulated by YY1. Further experiments conducted in CCLP1 and QBC939 cells showed that YY1 overexpression had no significant effect on PCIF1 expression.^64^ However, another study showed that the knockdown of YY1 in cholangiocarcinoma cell lines CCLP1 and RBE resulted in a decrease in PCIF1 expression at both the translational and transcriptional levels. Knocking down of PCIF1 inhibited the proliferation and migration of CCLP1 and RBE cells.^65^

Indeed, the m6Am level in serum was also markedly higher in colorectal cancer (CRC) than in healthy controls. The area under the curve was 0.791, suggesting that m6Am was a better marker for CRC than for gastric cancer.^62^ In CRC, PCIF1 mRNA and protein expression are higher in both CRC tissues and CRC cells than in their normal counterparts. The PCIF1 staining intensity was not associated with tumor stage, pathology, or grade, but high PCIF1 expression was linked to poor survival. PCIF1 accelerated CRC cell proliferation, invasion, and migration, as well as fibronectin adhesion and colony formation by controlling the stability of FOS, a proto-oncogene involved in the transforming growth factor beta (TGF-β) pathway, through an m6Am-dependent mechanism. The TGF-β signaling pathway has been increasingly recognized as a key factor in tumor development and progression and in the immunosuppressive tumor microenvironment,^66^^,^^67^ and it has a well-established role in CRC.^68^

**Figure 4:**
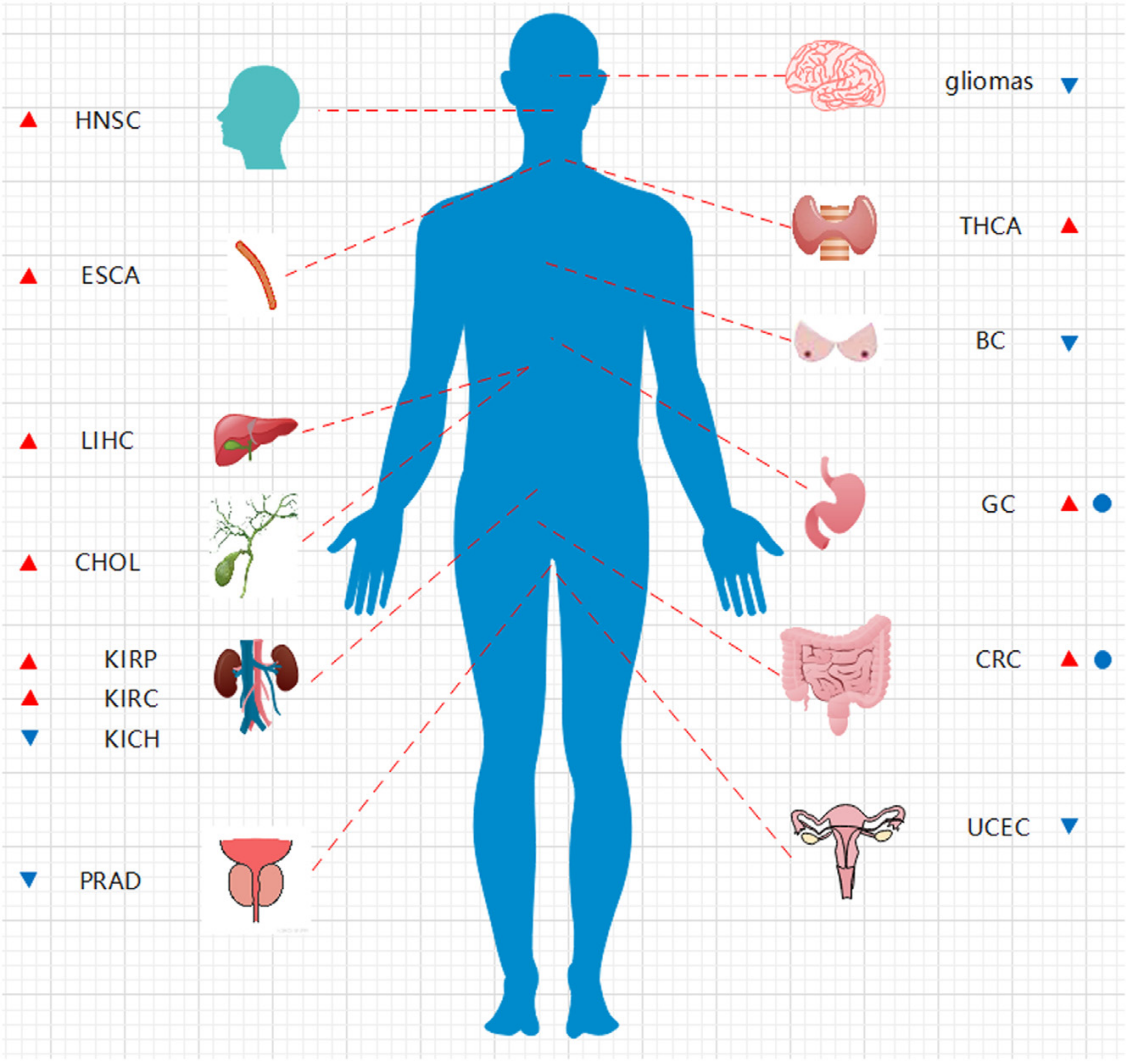
Dysregulation and survival effect of PCIF1 in different cancers. The red triangle indicates higher PCIF1 levels, while the blue triangle represents lower PCIF1 levels. The blue circle indicates that higher PCIF1 predicts worse survival. HNSC, head and neck squamous cell carcinoma; ESCA, esophageal carcinoma; LIHC, hepatocellular carcinoma; CHOL, bile duct cancer; KIRP, kidney papillary cell carcinoma; KIRC, kidney clear cell carcinoma; KICH, kidney chromophobe; PRAD, prostate cancer; THCA, thyroid cancer; BC, breast cancer; GC, gastric cancer; CRC, colorectal cancer; UCEC, endometrioid cancer.

In recent research, PCIF1 expression was found to be significantly elevated, and PCIF1 seemed to play as an oncogene in digestive system tumors, including gastric cancer, CRC, esophageal carcinoma, and hepatocellular carcinoma.^56^^,^^57^ However, PCIF1 exhibited significantly decreased expression and served as a tumor suppressor in other tumors, such as prostate cancer, breast cancer, and thyroid cancer (Fig. 4; Table 2).^56^^,^^57^ PCIF1 has also been found to suppress the proliferation of bladder cancer^60^ and glioma cells.^69^ PCIF1 inhibits glioma cell proliferation both *in vitro* and *in vivo*, while its knockdown increases cell proliferation and cell cycle progression and reduces apoptosis. However, the anti-tumor role of PCIF1 is not fully dependent on its methyltransferase activity. Overexpressing APPA mutant also significantly decreased the glioma cell viability. Though significant promoted cell proliferation can be observed in immortalized glioma cell line U251 when PCIF1 is significantly down-regulated, m6Am/A level has not been affected. Moreover, PCIF1 overexpression causes cell cycle arrest at the G2/M phase and apoptosis in glioma cells. Thus, the specific role of PCIF1 in cancer progression may vary depending on the cell or tissue type, which needs further investigation in other tumors. The physiological and pathological functions may not only rely on its methyltransferase activity but also involve other non-methyltransferase activities.

**Table 2:** The role of PCIF1 in various cancers.

Cancer	PCIF1 level	Role	Prognosis	Functions	PCIF function	Mechanism	Target/pathway	Refs
gastric cancer	Up	oncogenic function	poor	1 cell proliferation 2 cell invasion 3 correlate with stages	methyltransferase activity	mRNA translation	PCIF1—TM9SF1	^56^
gliomas	Down	cancer suppressor	No correlation	1 cell proliferation 2 cell cycle progression block 3 cell apoptosis	methyltransferase activity and non-methyltransferase activity	/	/	^69^
colorectal cancer	Up	oncogenic function	poor	1 cell proliferation 2 cell invasion 3 cell migration and fibronectin adhesion 4 colony formation 5 did not correlate with stage, pathology grade.	methyltransferase activity	mRNA stability	1.PCIF1-FOS-TGF-β 2.PCIF1-Stat1/Ifitm3-IFN-γ	^57^

Interestingly, PCIF1 has been shown to have opposing physiological functions in different types of cells and even in the same cell line. The conflicting roles of PCIF1 reported might be partially explained by the different detection methods, experimental conditions, and other potential biases in the studies. A comprehensive understanding of the roles of PCIF1 in different types of cancers is essential for guiding therapeutic interventions.

PCIF1 affects the response of CRC to anti-PD-1/PD-L1 therapies, which are effective in advanced rectal cancer.^70^^,^^71^ PCIF1 influences two pathways that are important for the efficacy of immunotherapy: the TGF-β pathway and the IFN-γ pathway in CRC. PCIF1 modifies the mRNA of Fos, which is involved in the TGF-β pathway. TGF-β can reduce the efficacy of anti-PD-1/PD-L1 therapies by changing the tumor microenvironment and helping tumor cells escape immune attack.^72^ TGF-β has also been found to be a predictor of the response to anti-PD-1/PD-L1 therapies in gastrointestinal tumors.^73^ PCIF1 also regulates m6Am modification of Ifitm3 and Stat1, which are involved in the IFN-γ pathway. IFN-γ can enhance the efficacy of anti-PD-1/PD-L1 therapies by activating immune cells and killing tumor cells. PCIF1 knockdown makes CRC tumors more sensitive to anti-PD-1 therapy by increasing the population of natural killer cells and decreasing that of monocytic myeloid-derived suppressor cells in the tumor microenvironment. PCIF1 plays a role in immunotherapy resistance in CRC by modifying the m6Am landscape in mRNA and affecting the tumor–immune system interactions.

The role of PCIF1 in facilitating cancer development suggests that targeting this modification could have beneficial effects on cancer treatment. PCIF1 could be a promising therapeutic target for both directly suppressing cancer development and modulating the response to immunotherapy, as physiological conditions, viability, and fertility are not affected by PCIF1 knockdown.^10^^,^^33^ Therefore, we anticipate that controlling PCIF1 expression could suppress cancer progress and increase the efficacy of immunotherapy by controlling m6Am modification of target genes. Likewise, inhibitors of METTL3/METTL14 methyltransferase that inhibit acute myeloid leukemia growth and decrease the population of leukemia stem cells *in vivo* have been recently developed to treat myeloid leukemia.^74^ Several improved FTO inhibitors have also been developed since 2020, such as CS1/CS2^75^ and Dac51,^76^ which suppress cancer cell proliferation and cancer stem cell self-renewal as well as enhance anti-tumor immunity. Wang et al^57^ developed a lipid nanoparticle-mediated siRNA delivery system. After injection of the lipid nanoparticle-PCIF1 siRNAs formulation into the athymic nude mice with CRC, efficient silencing of PCIF1 in the tumor was observed and the tumor size was significantly reduced. Both the direct inhibition of PCIF1 expression through siRNA therapy and the inhibition of PCIF1 methyltransferase activity through structurally designed small molecule compounds suggest new possibilities to improve therapeutic strategies for patients with cancer. Consequently, PCIF1 could be a promising and feasible therapeutic target for cancer.

## PCIF1 and viral infection

RNA transcripts can undergo modifications that influence viral replication through various mechanisms involving viral RNA transcription, splicing, stability, or export.^77^, ^78^, ^79^, ^80^ RNA modifications have important roles in modulating interactions between host and virus. m6Am RNA modification and its methyltransferase involved in biological and disease processes, especially in viral infections and host–pathogens interactions, have received increasing attention.

Modifications of viral RNAs can be mediated by enzymes encoded by the viral genome or by enzymes hijacked from the host cell.^81^ It has been demonstrated that modifications of both viral and host RNAs can affect the interactions between the virus and the host.^82^, ^83^, ^84^, ^85^ With the introduction of analytical methods and technological innovations for the study of m6Am, the number of studies focused on m6Am RNA modification and the catalytic functions of PCIF1 has greatly increased. Viruses have evolved various mechanisms to facilitate their replication in host cells. First virus can reprogram host cellular RNA m6Am methylome to facilitate its replication. HIV replication is restricted by host PCIF1 in a manner dependent on its methyltransferase activity.^59^ Unlike other viruses such as vesicular stomatitis virus,^86^ rabies virus, measles virus,^81^^,^^86^ and Kaposi's sarcoma-associated herpesvirus,^77^ which have PCIF1-mediated m6Am modifications in their mRNA cap structure, HIV transcripts do not have PCIF1-mediated m6Am modifications in their mRNA cap structure.^86^ PCIF1 inhibits HIV infection by directly binding to host transcripts and stabilizing m6Am-modified host genes such as ETS1, which suppresses HIV transcription by binding to the HIV promoter. However, the virus does not simply wait to die. HIV counteracts this inhibition by targeting PCIF1 with its viral protein R which triggers the ubiquitination and degradation of PCIF1.

Various coronaviruses, such as SARS-CoV, MERS-CoV, and SARS-CoV-2 and its variants of concern like Delta, Beta, and Omicron, can infect cells more easily with the help of PCIF1. PCIF1 increases the mRNA stability of angiotensin-converting enzyme 2 and transmembrane serine protease 2, which are the key entry receptors for these viruses, via its m6Am methyltransferase activity.^58^ On the other hand, PCIF1 does not affect infection with vesicular stomatitis virus, which uses low-density lipoprotein receptors for entry. PCIF1 knockout in Calu-3 cells results in the down-regulation of 172 genes involved mainly in pathways related to inflammatory response, cell adhesion, and phosphoinositide 3-kinase-AKT-mammalian target of rapamycin signaling. The SARS-CoV-2 and COVID-19 pathway, which includes angiotensin-converting enzyme 2 and transmembrane serine protease 2, is the pathway with the second-highest enrichment in these genes. PCIF1 promotes SARS-CoV-2 infection by keeping angiotensin-converting enzyme 2 and transmembrane serine protease 2 mRNA stable through m6Am methylation.

Another important way of regulating viral–host interactions is m6Am modification of viral RNAs. PCIF1 can deposit methyl groups on both vesicular stomatitis virus and rabies virus mRNAs at their m6Am sites, but this does not influence the stability, translation, replication, or expression of these RNAs under physiological conditions. However, when cells are pre-treated with interferon-β, m6Am modification of viral RNAs weakens the anti-viral effect of interferon-β. This weakened anti-viral effect is not dependent on the sensing of retinoic acid-inducible gene I or interferon-induced protein with tetratricopeptide repeats-1, suggesting that other strategies of viral detection or other mechanisms may be involved in the reduction in the host innate immune response due to m6Am modification of viral RNAs.

The recognition of the involvement of PCIF1 and m6Am modification in virus–host interactions, suggests that PCIF1 may be a promising therapeutic target for viral infections. The roles of PCIF1 in different viral infections are shown in Table 3.

**Table 3:** The role of PCIF1 in viral infection.

Infection virus	PCIF1 function	Viral target	Host target	Mechanism	Effect on viral	Refs
HIV	methyltransferase activity	N	ETS1	enhance mRNA stability	Repress viral replication	^59^
VSV	methyltransferase activity	pan-target	/		m6Am methylation of viral mRNAs protects against the otherwise antiviral effects of the IFN-mediated innate immune response	^86^
SARS-CoV-2	methyltransferase activity	/	ACE2 TMPRSS2	enhance mRNA stability	Facilitates SARS-CoV-2 entry	^58^

## Conclusion

Since the discovery of PCIF1 as the specific m6Am methyltransferase for mRNA cap structures in 2018, considerable efforts have been made to elucidate the role of the m6Am modification and its machinery in various aspects of mRNA metabolism, cellular processes, and disease pathogenesis. It has become evident that m6Am modification affects the expression of multiple genes. However, the current annotations of m6Am and m6A in the transcriptome may be inaccurate, as the original m6A-seq mapping technique cannot distinguish between m6A and m6Am (m6A/m),^25^ and heterogeneity of transcription-start sites may also contribute to the mis-annotations.^15^ Therefore, some of the biological effects attributed to m6A modification may be partially mediated by m6Am modification.

PCIF1 and its catalyzed m6Am modification have been implicated in various diseases, such as glucose homeostasis disorders,^10^ viral infections,^11^ obesity,^12^ and cancers.^13^ These findings highlight the essential roles of PCIF1 in disease development and progression and suggest a potential value of targeting PCIF1 for disease treatment. However, the impact of PCIF1 and m6Am on gene expression and their correlation with disease correlation are still largely unexplored. As the fact that PCIF1 as a cap-specific m6Am methyltransferase has only recently been identified, research on PCIF1 and m6Am is limited. Currently, the established studies have focused mainly on the internal m6A and its modification system. However, the m6A modification system is complicated and contains multiple writers, erasers, and readers. In contrast, PCIF1 is thus far the only identified cap-specific m6Am methyltransferase, thus constituting a more precise target for disease prediction or intervention. One of the major limitations in m6Am study may be the methodologies for m6Am detection. Most extant studies utilized nonspecific methodologies and caused controversial results. Hopefully, more and more specific and accurate methodologies have been applied. Further studies are warranted to fully elucidate the roles and underlying molecular mechanisms of PCIF1 in-depth, and these findings are expected to provide critical information for future clinical interventions.

PCIF1 plays a crucial role in various diseases and targeting PCIF1 may offer a new way to treat them. However, our knowledge of how PCIF1 and m6Am affect gene expression and disease association remains limited. More in-depth studies are needed to fully understand the functions and molecular mechanisms of PCIF1, knowledge expected to help us develop better clinical interventions.

## Author contributions

HZ drafted the manuscript, YW reviewed the manuscript, XL conceived the study, and all the authors read and approved the final manuscript.

## Conflict of interests

The authors declare no conflict of interests.
